# Metastatic Clivus Chordoma: A Case of a Rare Tumor in a 29-Year-Old African American Male

**DOI:** 10.7759/cureus.21694

**Published:** 2022-01-28

**Authors:** Olga Lopez, Amro Al Ashi, Guillermo Izquierdo-Pretel

**Affiliations:** 1 Medicine, Florida International University, Herbert Wertheim College of Medicine, Miami, USA; 2 Internal Medicine, Florida International University, Herbert Wertheim College of Medicine, Miami, USA

**Keywords:** signaling pathways, palliative, radiation, african american, oncology, metastasis, clivus chordoma

## Abstract

In this study, we report a case of a late-stage clival chordoma in a 29-year-old African American male and the unfortunate linear progression course since his initial diagnosis. Upon his initial encounter in 2020, radiation therapy did not offer any promising curative outcome. He was initially treated with a combination treatment of partial resection, radiation, and proposed oral imatinib, none of which modified the natural history and progression of his illness. Instead, these methods were performed as palliative measures to reduce the current size of the tumors and decrease growth rates to minimize his pain. Social issues acted as a contributory risk factor in his prognosis and due to the patient's socioeconomic barriers, he was not able to continue seeking available radiotherapy, leading to disease exacerbation. Poor adherence was noted with his follow-up. The risks of being affected by this disease are likely multifactorial and more reports of such cases need to be added to bridge this gap in the current literature. In addition, there is a gap in the current study of reports of such tumors found in diverse racial groups and in patients who are in their first few decades of life. Novel treatment strategies were reviewed, and it is expected they could generate assertive treatment guidelines.

## Introduction

Chordomas are rare occurring slow-growing tumors with a tendency for the local invasion that have poor treatment guidelines [1-4]. Chordomas are derived from embryonic origin of the remnants of the notochord, specifically between the clivus and sacrum. These tumors most commonly affect the sacrum, base of skull, and vertebral bodies of the spine and account for up to 4% of all malignant bone tumors [1-4]. The annual incidence of chordomas is 1:100,000 for new diagnoses, in addition to 300 new cases in the United States affecting more males than females with ratio of 2:1 [2-4]. Clival chordomas are a rare bone cancer affecting <1 per 100,000 and are an embryological derivative of the notochord [1].

The pathophysiology of chordomas is still not well understood, but it has been associated with several signaling pathways including *mTOR*, *PTEN *gene deficiency, *c-MET*, *INI-1*, and *PDGFR-ß *[4]. This invasive and highly metastatic cancer most commonly affects patients between the age of 35 and 60 years and rarely affects patients in the first few decades of life [1]. In this case presentation, we add to the current literature of reported chordoma cases by presenting a rare onset of metastatic clival chordoma in a 29-year-old African American male. Clival chordoma is a subtype of chordoma that arises at the base of skull with metastatic potential involving the spine. Due to the rarity of the disease, no clear guidelines have been established to treat or manage patients with this condition. From this case report, we hope to increase awareness about this devastating type of cancer in diverse racial groups and the necessity to advocate for patients and for the development and implementation of treatment and pain management guidelines with the goal of potential to cure cancer, increase survival rates, and/or provide patients with pain alleviation and comfort.

## Case presentation

A 29-year-old African American male with multiple socioeconomic barriers including substance use disorder, uninsured status, unemployment, and previous incarceration presented to in-patient hospitalist service with concerns of worsening neck and back pain related to his clival chordoma. On examination, the patient was normotensive with a blood pressure of 120/78 mmHg, and a heart rate of 103 bpm. The patient was afebrile with a temperature of 36.9⁰C. He was coronavirus disease 2019 (COVID-19) negative, herpes simplex virus 1 and 2 positive, and had a hoarse voice. On dermatologic examination, he had scattered maculopapular lesions throughout the abdominal and back area. Pressure ulcers on the sacral regions bilaterally were present. 

He initially presented in 2019 with chief complaints of occipital headaches and at that time, he was diagnosed with a clival chordoma followed by partial resection and adjuvant radiation. Later in the same year, he had subsequent hospitalizations in which surgical intervention was offered to him but due to high morbidity of procedures, the patient refused any further surgical intervention. 

In December 2020, the patient presented to the emergency department with hearing loss in the left ear, left eye vision loss, and dysphagia and was further treated with radiation to the skull base and cervical spine (20 Gy out of planned 25 Gy in four of five fractions). 

In May 2021, he presented again to the emergency department with two weeks of right lower extremity weakness, paresthesias, and decreased sensation. He also reported pain in his right lower back/hip region associated with paresthesias radiating down his leg. During this same admission, drop metastases in his thoracic and lumbar spine were discovered. The care team planned for the patient to receive 30 Gy in 10 fractions; however, he could not return to complete his treatment as planned due to transportation issues. Oncology was consulted at this time and next-generation sequencing results were positive only for* KRAS* mutation, and a decision was made to start imatinib 400 mg two times daily.

**Figure 1:**
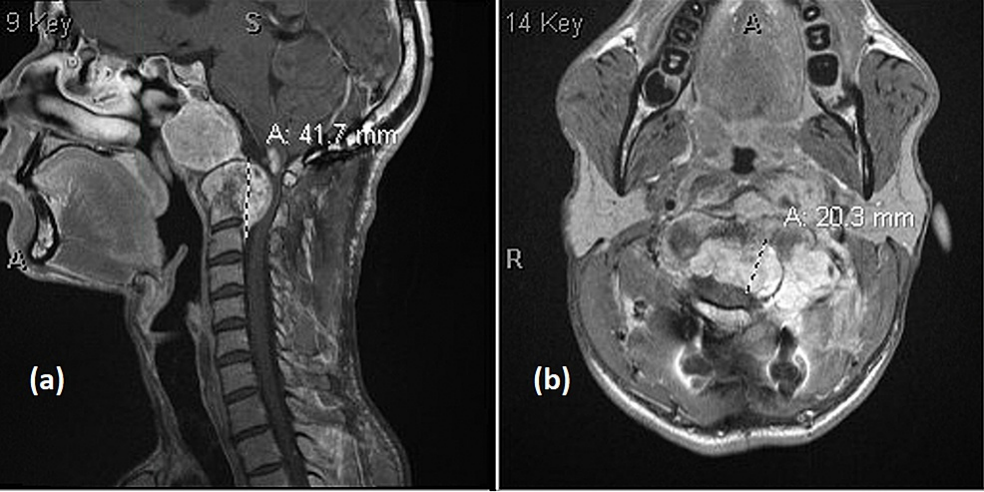
MRI spine of (a) large clival and (b) skull base chordoma. The image is showing large clival and skull base chordoma involving C1 and C2 with intraspinal epidural extension down to C3 and involvement of the left greater than right C1-C2 neural foramina. The mass effect on the cervicomedullary junction is greater in the left ventral cord than in the right.

**Figure 2:**
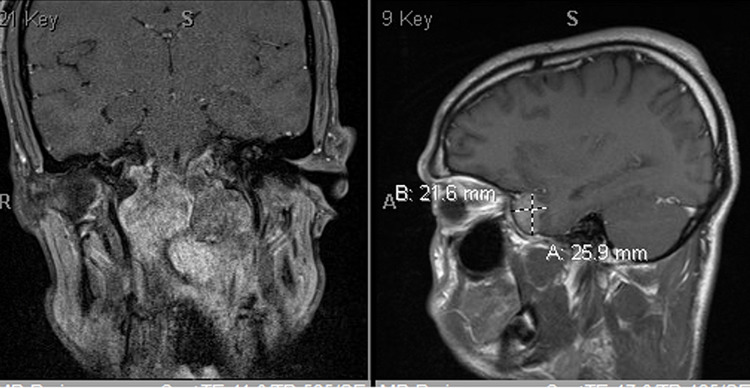
Extensive local invasion with mass effect on numerous skull base, brainstem, and posterior fossa structures as detailed above. Mass effect in the posterior fossa resulting in effacement of the fourth ventricle and cervicomedullary junction as well as cerebellar tonsillar herniation. A metastasis along the anterior aspect of the right temporal lobe/right sphenoid.

**Figure 3:**
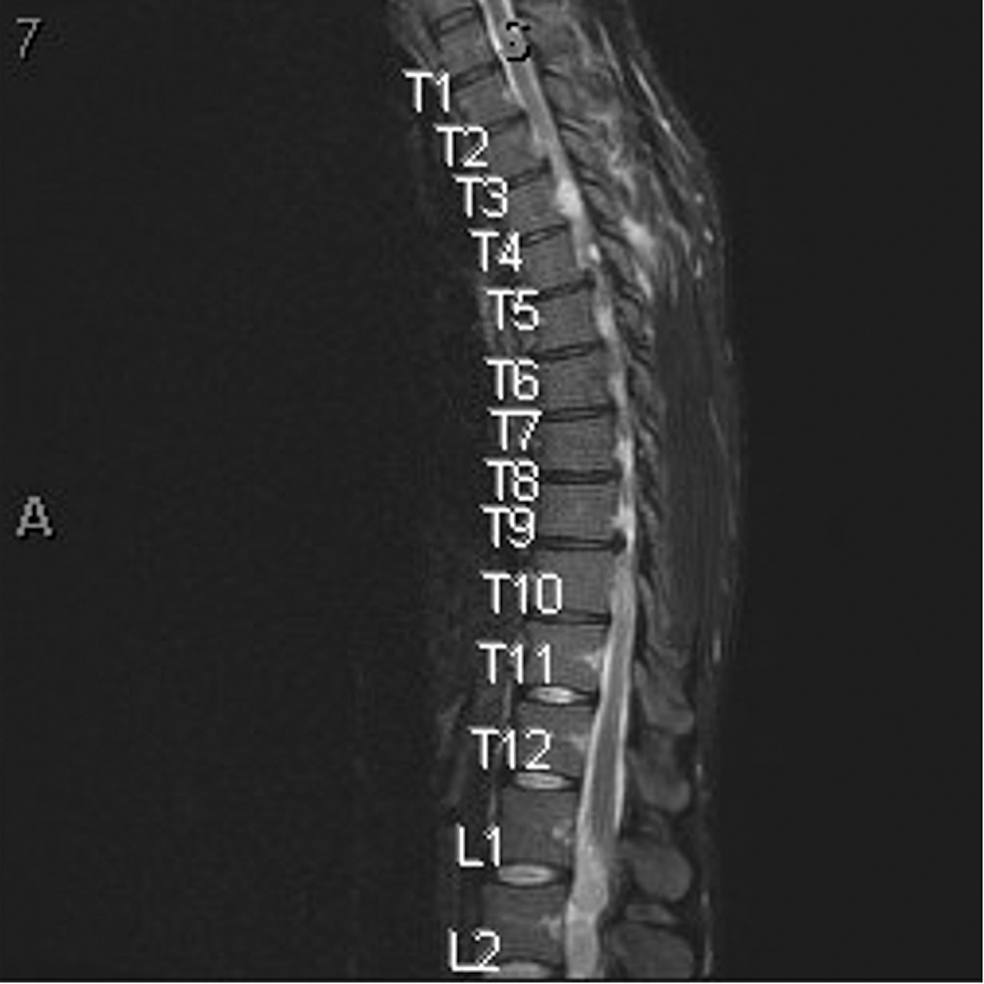
Multiple intradural drop metastases in the thoracic levels related to the patient's known malignant chordoma, worse at the T2-T3 level.

**Figure 4:**
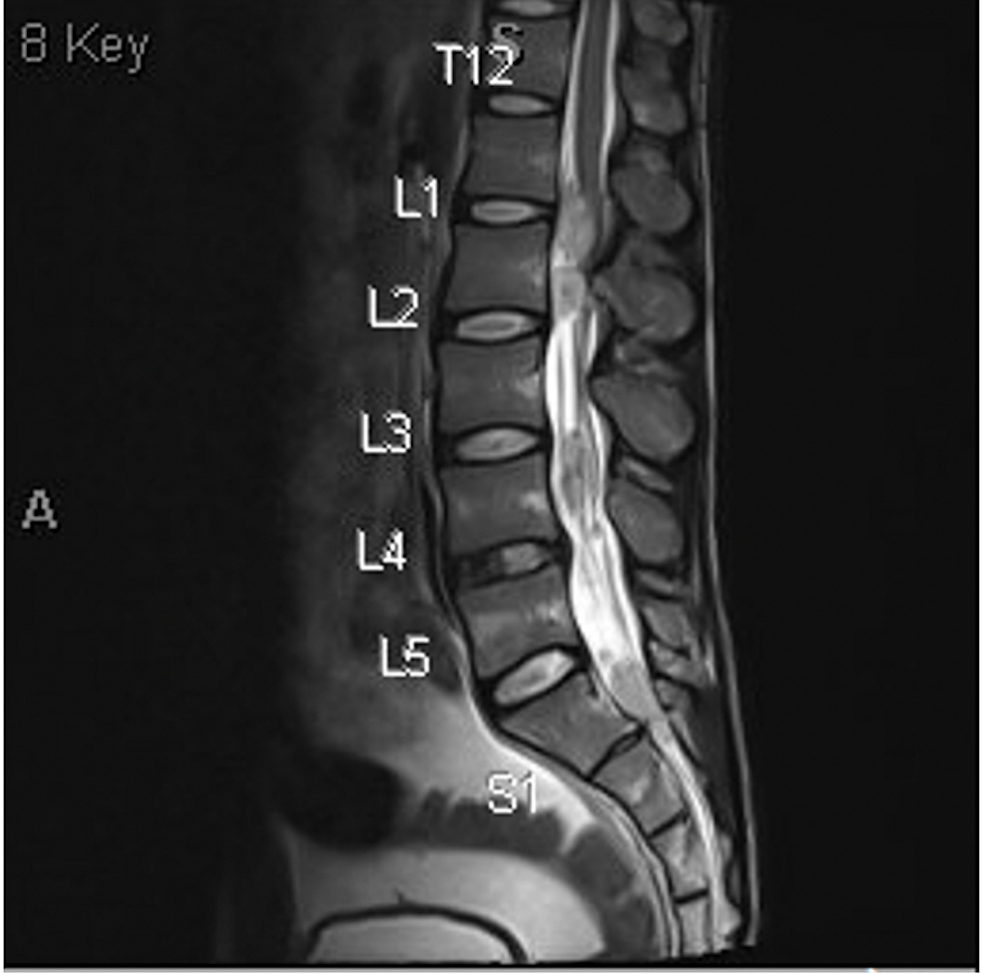
Numerous intradural drop metastases causing severe spinal canal stenosis with compression of the distal spinal cord and cauda equina nerve roots.

The patient presented to the emergency department again later in the month with similar symptoms of pain, progressive leg weakness, urinary retention, and bowel incontinence. MRI lumbar spine on May 30, 2021, confirmed interval worsening of his disease. He completed another course of radiation and was discharged on June 9, 2021, but returned shortly with further progression of his symptoms. A repeat MRI spine was done and remonstration of extensive intradural, extramedullary, enhancing masses along the imaged portion of the distal spinal cord and cauda equina nerve roots, consistent with intradural drop metastases from the patient's known malignant chordoma (Figures 1, 2), with essentially complete obliteration of the thecal sac at L1-L2, L2-L3 and L4 (Figures 3, 4). 

Oncology was consulted for recommendations on all hospitalizations, and he was last evaluated by the oncological team on June 30, 2021, after his case was discussed by the neuro-oncology tumor board. The recommendation was to continue imatinib for an additional month and then repeat imaging to determine if the patient has responded. If no response, per discussion with the tumor board, the next best option for therapy would be erlotinib. The patient declined treatment, signed a “do not resuscitate” order, and decided to pursue hospice. He was discharged to an in-patient hospice unit on July 1, 2021. 

## Discussion

Clival chordomas are a rare bone cancer affecting <1 per 100,000 and are an embryological derivative of the notochord [1]. Considering their invasive nature, the 10-year survival rate for patients with chordomas is estimated at around 50%, with complete resection of the tumor followed by chemotherapy [2,3]. On the contrary, the 10-year survival rate for patients with clival chordoma, a rare variant of chordomas, was estimated at 60% following surgical resection and radiotherapy given that combined treatment modalities are preferred [4-6]. Chordomas tend to affect males at a rate double that of females and tend to present after the first few decades of life [7-9]. However, the age at which chordomas present heavily depends on the race and the genetic makeup of the individual. In a comparative study by Parry et al., using the surveillance, epidemiology, and end results (SEER) database, there is evidence to show that the average age for a chordoma to affect a patient that has a high-risk familial genetic predisposition to chordomas was 26.8 years old while a matched patient with no genetic predisposition was 46 years old. The race was a pertinent finding as well given that the group that was deemed to be the highest risk for a chordoma occurrence were African Americans followed by Asian/Pacific Islander/American Indian/Alaska Natives and then White/Caucasian as having the lowest risk [7-9]. 

The most common diagnostic modality of such tumors is an MRI, once symptoms present given the indolent nature of these tumors. There are limitations in imaging such tumors given that general protocols for neurological imaging such as MRI’s often do not extend below the S2 level below which a large percentage of sacrococcygeal chordomas present [7,8].

The rate of removal of a chordoma tumor is higher in the initial phase of resection, with a progressive decline with subsequent resections [8-10]. The determination of the tier of initial treatment is determined by the severity of the tumor extension at initial presentation. Target treatment of such tumors is dependent largely on their immunohistochemical staining and makeup given that many of these tumors upon examination express tumor markers such as *PDGFR-α*, *EGFR,* and *c-MET* with a higher expression of *PDGFR-α* and *c-MET* in younger patients [5,10]. Those with *c-MET *expression tend to have a better prognosis compared to their *PDGFR-α* counterparts [5,8-10]. 

Current treatment strategies are multimodal and heavily targeted towards resection rather than medical management and tend to favor symptomatic management rather than curative intent [10]. Current ongoing studies are investigating the potential for the use of imatinib and everolimus for advanced chordoma treatment; however, the results of these studies are still pending [6]. Other studies are looking at minimally invasive endonasal techniques to target centrally located lesions which have traditionally posed a greater challenge for remission rates of affected patients [5,10].

## Conclusions

Considering our patient’s disease, racial background, age, and severe presentation, we hope to raise more awareness about the need to address the gap in the literature regarding such occurrences. Molecular target treatment based on immunohistochemical staining and diagnostic features could assist future treatment of such cancers even if only to provide a better quality of life and symptomatic relief when no curative attempt is possible. Given that younger age, the rarity of the subtype of this cancer, race, and socioeconomic background are all important factors to consider when treating patients with this specific pathology, we hope this case presentation draws attention to the need for more clearly defined guidelines for clival chordomas in such populations given the lack of such information in the current study.
